# Prevalence, genetic identity and vertical transmission of *Babesia microti* in three naturally infected species of vole, *Microtus* spp. (Cricetidae)

**DOI:** 10.1186/s13071-017-2007-x

**Published:** 2017-02-06

**Authors:** Katarzyna Tołkacz, Małgorzata Bednarska, Mohammed Alsarraf, Dorota Dwużnik, Maciej Grzybek, Renata Welc-Falęciak, Jerzy M. Behnke, Anna Bajer

**Affiliations:** 1 Department of Parasitology, Institute of Zoology, Faculty of Biology, University of Warsaw, 1 Miecznikowa Street, 02-096 Warsaw, Poland; 2 Department of Parasitology and Invasive Diseases, Faculty of Veterinary Medicine, University of Life Sciences in Lublin, 12 Akademicka Street, 20-950 Lublin, Poland; 3 School of Life Sciences, University of Nottingham, University Park, Nottingham, NG7 2RD UK

**Keywords:** *Babesia microti*, Prevalence, Genotyping, Vertical transmission, Congenital infection, Voles, *Microtus*

## Abstract

**Background:**

Vertical transmission is one of the transmission routes for *Babesia microti*, the causative agent of the zoonotic disease, babesiosis. Congenital *Babesia* invasions have been recorded in laboratory mice, dogs and humans. The aim of our study was to determine if vertical transmission of *B. microti* occurs in naturally-infected reservoir hosts of the genus *Microtus*.

**Methods:**

We sampled 124 common voles, *Microtus arvalis*; 76 root voles, *M. oeconomus* and 17 field voles, *M. agrestis.* In total, 113 embryos were isolated from 20 pregnant females. Another 11 pregnant females were kept in the animal house at the field station in Urwitałt until they had given birth and weaned their pups (*n* = 62). Blood smears and/or PCR targeting the 550 bp *18S rRNA* gene fragment were used for the detection of *B. microti*. Selected PCR products, including isolates from females/dams and their embryos/pups, were sequenced.

**Results:**

Positive PCR reactions were obtained for 41% (89/217) of the wild-caught voles. The highest prevalence of *B. microti* was recorded in *M. arvalis* (56/124; 45.2%), then in *M. oeconomus* (30/76; 39.5%) and the lowest in *M. agrestis* (3/17; 17.7%). *Babesia microti* DNA was detected in 61.4% (27/44) of pregnant females. Vertical transmission was confirmed in 81% (61/75) of the embryos recovered from *Babesia*-positive wild-caught pregnant females. The DNA of *B. microti* was detected in the hearts, lungs and livers of embryos from 98% of *M. arvalis*, 46% of *M. oeconomus* and 0% of *M. agrestis* embryos from *Babesia*-positive females. Of the pups born in captivity, 90% were born to *Babesia*-positive dams. *Babesia microti* DNA was detected in 70% (35/50) of *M. arvalis* and 83% (5/6) of *M. oeconomus* pups. Congenitally acquired infections had no impact on the survival of pups over a 3-week period *post partum*. Among 97 *B. microti* sequences, two genotypes were found. The IRU1 genotype (Jena-like) was dominant in wild-caught voles (49/53; 92%), pregnant females (9/11; 82%) and dams (3/5; 60%). The IRU2 genotype (Munich-like) was dominant among *B. microti* positive embryos (20/27; 74%) and pups (12/17; 71%).

**Conclusion:**

A high rate of vertical transmission of the two main rodent genotypes of *B. microti* was confirmed in two species of naturally infected voles, *M. arvalis* and *M. oeconomus*.

## Background

Voles of the genus *Microtus* constitute the main natural hosts of the protozoan parasite *Babesia microti* [1, 2]. In Poland, the highest recorded prevalence of *B. microti* is from the common vole (35–72% in *M. arvalis*), and then from the root vole (32–50% in *M. oeconomus*) [2–4]. There are few data for the less-well studied field vole, *M. agrestis* [3].

*Babesia microti* is an important zoonotic parasite, responsible for the great majority of human cases of babesiosis reported in the USA [5, 6]. In contrast, far fewer cases of human *B. microti* infections have been reported to date in Europe [7–9], although strains known to be pathogenic for humans have been isolated from common and root voles in north-eastern Poland [2].

Ticks of the genus *Ixodes* are the main vectors of *Babesia* parasites, with the common tick, *Ixodes ricinus*, being the main vector in Europe [10–12]. Prevalence in ticks is usually low (1–10%). *Ixodes ricinus* instars feed mainly on woodland rodents such as *Myodes glareolus* and *Apodemus flavicollis* [13] and are less abundant on rodents inhabiting open grasslands, such as voles from the genus *Microtus*. However, *Microtus* spp. generally show high prevalence of *B. microti* despite low infestation by *I. ricinus* instars and low prevalence of *B. microti* in this tick species. A similar phenomenon has been recognized in a rodent community near Omsk, Russia [14], where 30–60% of *Myodes* and *Microtus* spp. voles were found to be infected with *B. microti* but no *B. microti* infection was detected in ticks collected from rodents and from the environment [14]. We hypothesized that the high prevalence of *B. microti* in *Microtus* spp. in our area is maintained by alternative routes of transmission, the most likely of which is vertical transmission from female voles to their offspring.

Vertical transmission of *B. microti* has been clearly demonstrated recently in BALB/c mice in our laboratory [15], with up to 100% success, and some cases of congenital babesiosis have been reported recently in the literature in dogs [16–18]. Congenital babesiosis has been recognized also in newborn human babies in the USA [19–21].

The aim of the current study was to test the hypothesis that vertical transmission of *B. microti* occurs in naturally infected voles. Accordingly, we first planned to determine the prevalence of *B. microti* in embryos dissected from naturally infected voles, thus completely eliminating the possibility of vector-borne transmission. Then, to eliminate the possibility that the tissues of the embryos may have been contaminated by maternal blood, despite all the precautions that had been taken, and to evaluate the impact of congenital infection on the survival of pups, we planned to maintain in captivity naturally infected pregnant female voles, completely deprived of ectoparasites, until a suitable period after parturition when individual sampling of the blood of the pups was possible. Thus we could assess the prevalence of congenitally transmitted *B. microti* infection in the pups.

## Methods

The study was conducted within the Mazury Lake District of north-eastern Poland (Urwitałt, near Mikołajki; 53°48'50.25"N, 21°39'7.17"E), within an extensive forest and old field system adjacent to Lakes Śniardwy and Łuknajno. At the time of the study, the long-abandoned, previously intensively cultivated fields in the study sites had succeeded to a mixed vegetation of scrub and long grass. Trap lines extended up the gentle hills (greatest elevation 5 m) from two small ponds, giving a gradation in physical conditions and vegetation: from marshland, submerged during rainy weather, to dry grassland. We trapped three species of voles in these different microhabitats: *M. arvalis* individuals on the dry upper sections of the hills; *M. oeconomus* in the belts of marshland around the ponds and *M. agrestis* in the intermediate zones. Trapping of rodents took place in summer (August and early September) in 2013 and 2014. Rodents were live trapped using mixed bait comprising fruit (apple), vegetables (carrot or cucumber) and grain. Two traps were set every 10 m along the trap lines at dusk, and checked and closed the following morning to prevent animals entering during daytime and to avoid losses from excessive heat from exposure of traps to direct sunlight. Traps were then re-baited and re-set on the following afternoon. Traps were also closed during periods of intensive rainfall. At each location trapping was continued for at least 5 consecutive nights. All captured voles were transported in their traps to the laboratory for inspection.

In 2013, the autopsies were carried out under terminal isoflurane anesthesia. Animals were weighed to the nearest gram, and total body length and tail length were measured in millimeters. Animals were allocated to three age classes (juveniles, young adults and adults), based on body weight and nose-to-anus length together with reproductive condition (scrotal, semi-scrotal or non-scrotal for males; lactating, pregnant or receptive for females) [1, 22]. Ectoparasites (ticks, fleas, mites) were removed using forceps and preserved in 99.8% methanol. A blood sample was taken from the heart for direct preparation of two thin blood smears and storage in 0.001 M EDTA (anticoagulant) for subsequent DNA extraction. The upper (maxilla) and lower (mandible) jawbones of autopsied individuals were inspected to confirm identity of the vole species based on the known dental formula for each, and especially to distinguish between juvenile individuals of *M. oeconomus* and *M. agrestis* [23].

Initially vole species were distinguished based on their appearance (fur colour: grey and yellowish hair with brighter belly and legs: *M. arvalis*; brown-reddish fur with dark belly and legs: *M. agrestis*; dark brown fur with dark belly and black legs: *M. oeconomus*), and on body weight and body measurements, as follows: (i) *M. arvalis*: mean weight 25.4 g; mean body length 10.4 cm; mean tail length 3.1 cm; (ii) *M. agrestis*: mean weight 27.2 g; mean body length 10.9 cm; mean tail length 3.4 cm; (iii) *M. oeconomus*: mean weight 36.6 g; mean body length 11.9 cm; mean tail length 4.6 cm. Finally, we confirmed the species identity of each individual by examination of the lower molars M_1_ and M_2_ and the second upper molar (M^2^) [23]. Embryos were isolated and frozen at a temperature of -20 °C.

In the summer of 2014, all the captured voles were live-processed. Voles were taken to the laboratory, where under non-terminal isoflurane anesthesia they were weighed to the nearest gram, and total body length and tail length were measured in millimeters. Data on age, sex and reproductive condition were recorded, and the ectoparasites (ticks, fleas, mites) carefully removed and preserved, as described above. A blood sample was taken from the tail tip of each vole (for blood smears and for preservation in EDTA to facilitate DNA extraction, as described above). Then males and juveniles were released in close proximity to the trap lines where earlier they had been caught. Females suspected of being pregnant were transferred to individual cages to establish a breeding colony of voles. The colony was maintained in the animal house at the field station in Urwitałt. Each cage contained a thick layer of standard sawdust (*c.*10 cm), water and food (grass, vegetables, fruits, grain) ad libitum together with bedding material (grass, hay, paper tubes). To prevent the development of ectoparasites (i.e. development of nymphs from engorged tick larvae), possible vectors, and to provide suitable housing conditions for pups, the cages were cleaned at least once a week. During handling, all voles from the breeding colony were inspected for ectoparasites in order to ensure vector-free conditions in the cages and animal house. No ectoparasites were noted at any time after initial caging, neither on the dams nor on the pups. Females were kept at a constant temperature of 18 °C, and with a 16 (Day): 8 (Night) light-dark phase for at least 3 weeks to allow pregnancies to develop to term. Non-pregnant females were then released at their original trap lines.

Pups were kept together with their dams for one month. In the third week of life we weighed the pups and collected blood samples from the tail tip of each individual. Then pups and dams were released at the trap lines at which the dams had been caught originally.

### Blood collection and DNA extraction

**Fig. 1:**
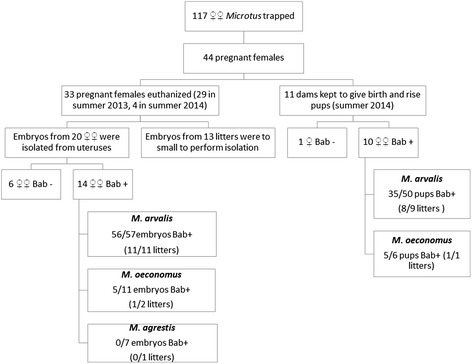
The scheme of the study. *Abbreviations*: Bab+, voles infected with *B. microti*; Bab-, voles uninfected with *B. microti*

Two thin blood smears were prepared from drops of blood taken from the heart (autopsies) or tail tip (alive processing) of wild-caught voles and pups. Blood smears were air-dried, fixed in absolute methanol and stained with Diff Quick (Microptic, Barcelona, Spain) and Hemacolor (Merck, Darmstadt, Germany) staining kits. Molecular techniques were used for the detection of *Babesia* in adult voles (males and females), embryos and pups*.* Between 20 μl (from the live-processed animals) to 200 μl of whole blood (from the culled animals) were collected into 0.001 M EDTA and frozen at a temperature of -20 °C before DNA extraction. Embryos were isolated from the uterus and individually processed (autopsies) following two washes in sterile water, to minimize contamination with maternal blood. We autopsied 113 embryos from 20 litters (16 obtained in 2013 and 4 litters from 2014 from females that succumbed under anesthesia) (Fig. 1). Organs (mainly hearts and lungs together, and brains, livers, spleens and kidneys, if distinguishable) were isolated from embryos with sterile dissecting instruments. Genomic DNA was extracted from whole blood and organs using the DNAeasy Blood & Tissue kit (Qiagen, USA) and stored at a temperature of -20 °C. The remaining 13 litters were in earlier stages of pregnancy (1–2 trimester) and were too small (diameter of the embryo together with amniotic sac less than 1 cm) to enable the isolation of specific internal organs.

### Molecular characterization

Detection and genotyping of *B. microti* isolates from pregnant females and embryos, dams and pups were performed by the amplification and sequencing of the 550 bp *18S rRNA* gene fragment by PCR (first run) and nested-PCR (in the case of no or weak signal from the initial one-step PCR). The primers and thermal profile used in this study have been described previously [24]. Reactions were performed in 1× PCR buffer, 1U Taq polymerase, 1 μM of each primer and 2 μl of the extracted DNA sample. Negative controls were performed in the absence of template DNA. In the PCR reaction, primers GF 5'-G(C/T)(C/T) TTG TAA TTG GAA TGA TGG-3' and GR 5'-CCA AAG ACT TTG ATT TCT CTC-3' were used for the amplification of a 559 bp fragment of *18S rDNA.* In the first step of nested-PCR, the full-length *18S rDNA* was amplified with apicomplexan 18S rRNA-specific primers: Crypto F (5'-AAC CTG GTT GAT CCT GCC AGT-3') and Crypto R (5'-GCT TGA TCC TTC TGC AGG TTC ACC TAC-3'). The PCR conditions included: 95 °C for 10 min, followed by 45 cycles of denaturation at 95 °C for 45 s, annealing at 60 °C for 45 s, and extension at 72 °C for 45 s. Final extension was at 72 °C for 7 min, followed by a hold step at 4 °C. In the second step (nested reaction), primers GR and GF were used. Nested PCR reactions were performed with different volumes of the first PCR product: 1 or 0.5 μl, or finally with 2 μl of the dilution 1: 9 in sterile water. As positive controls we used the genomic DNA of *B. microti* King’s 67 strain or *B. canis* DNA extracted from dog blood [25–27].

PCR products were subjected to electrophoresis on a 1.5% agarose gel, stained with Midori Green stain (Nippon Genetics, GmbH, Düren, Germany). Selected PCR products from voles trapped in 2013 and 2014, all pregnant females and dams, and from at least two pups per litter were sequenced by a private company (Genomed S.A., Gdańsk, Poland). DNA sequence alignments and analyses were conducted using MEGA v. 6.0. [28]. Consensus sequences were compared with sequences deposited in the GenBank database using BioEdit tool [29].

### Statistical analysis

The statistical approach adopted has been documented comprehensively in our earlier publications [30–33]. Prevalence (percentage of animals infected) was analysed by maximum likelihood techniques based on log-linear analysis of contingency tables. For analysis of the prevalence of *Babesia* in wild-caught voles, we fitted prevalence of *Babesia* infection as a binary factor (infected = 1, uninfected = 0) and then year (two levels: 2013, 2014), host species (three levels: *M. arvalis*, *M. oeconomus*, *M. agrestis*), host age (three levels: juvenile, young adult, adult) and host sex (two levels: males and females) as factors. Subsequent analyses were carried out for each host species separately, but without inclusion of ‘host species’.

For analysis of the prevalence of *Babesia* in embryos, we implemented ‘female infection’ as a binary factor (i.e. infected/uninfected mother). For analysis of the prevalence of *Babesia* in pups, we implemented pup survival as a binary factor (dead = 0 or alive = 1 at the age of 3 weeks). Beginning with the most complex model, involving all possible main effects and interactions, those combinations not contributing significantly to explanation of variation in the data were eliminated stepwise, beginning with the highest-level interaction. A minimum sufficient model was then obtained, for which the likelihood ratio of *χ* ^2^ was not significant, indicating that the model was sufficient in explaining the data.

Statistical analysis was carried out using SPSS v. 21.0. Multifactorial analysis of variance (ANOVA) was used for comparison of mean parameters (abundance of *B. microti*, litter size, mean weight of pup, etc.), which are reported with standard errors of their means (SE). Abundance of *B. microti* infection was calculated as the number of infected red blood cells (iRBC) in 200 fields of vision (×1,000 magnification). When samples were only positive by PCR, an intensity of 0.001 iRBC/200 fields was implemented into quantitative statistical analysis. Fisher’s exact test (INSTAT software) was used to compare the % of infected pups between *Babesia*-negative and *Babesia*-positive females.

The success of vertical transmission to each litter, calculated as the % of *Babesia*-positive pups/litter, was correlated with the litter size using the Spearman’s rank correlation test (SPSS v. 21).

## Results

### Prevalence of *B. microti* in the community of voles

#### Community structure

The number of wild-caught voles by year of study, host species, age and sex is provided in Table 1. In total, 217 voles of three species were trapped and sampled: 124 common voles, *M. arvalis*; 76 root voles, *M. oeconomus* and 17 field voles, *M. agrestis*. Adult individuals constituted the majority of the sampled vole community (70%), followed by young adults (18%); juveniles (12%) were least frequent. Females were slightly more abundant than males (54 *vs* 46%).

**Table 1:** Wild-caught *Microtus* voles sampled in 2013–2014

		2013				2014				
Vole species		Age class				Age class				
	Sex	1	2	3	2013 Total by species	1	2	3	2014 Total by species	TOTAL
*M. agrestis*	♂	0	2	7	14	0	0	1	3	17
	♀	1	2	2		0	2	0		
	♂ + ♀	1	4	9		0	2	1		
*M. arvalis*	♂	2	1	17	55	5	1	27	69	124
	♀	5	11	19		4	6	26		
	♂ + ♀	7	12	36		9	7	53		
*M. oeconomus*	♂	0	1	11	19	7	1	17	57	76
	♀	1	3	3		2	9	21		
	♂ + ♀	1	4	14		9	10	38		
Total by age class		9	20	59	88	18	19	92	129	217

*Abbreviations*: age class 1, juvenile; 2, young adult; 3, mature

#### Prevalence of *B. microti* in voles

Prevalence of *B. microti* infection by year of study, host species and sex is provided in Table 2. In total, a positive product of the specific PCR reaction was obtained for 41% of voles in the community. The highest prevalence of *B. microti* was detected in *M. arvalis* and the lowest in *M. agrestis* (presence/absence of *Babesia* × host species: *χ* ^2^ = 5.84, *df* = 2, *P* = 0.054). Prevalence increased significantly with the age of a host (presence/absence of *Babesia* × age class: *χ* ^2^ = 20.36, *df* = 2, *P* < 0.001). Overall, prevalence of *B. microti* was higher in males than females (47 *vs* 36%) but this difference was not statistically significant, and there were no differences in prevalence between the years of study (Table 2). However, there were significant differences in the pattern of infections among male and female voles in the community over the two years of the study (year × host sex × presence/absence of *Babesia*: *χ* ^2^ = 6.34, *df* = 1, *P* = 0.012). In 2013 prevalence of *B. microti* infection was similar in females and males (44.7 and 41.5%, respectively) while in 2014 prevalence was markedly higher in males in comparison to females (51 *vs* 30%, *P* = 0.012).

**Table 2:** Prevalence of *Babesia microti* in three species of wild-caught *Microtus* voles

Year		*M. arvalis*			*M. agrestis*			*M. oeconomus*			*Microtus* spp.		
	Infection	♂	♀	All	♂	♀	All	♂	♀	All	♂	♀	Total
2013	NI	11	18 (9)	29	7	4 (2)	11	6	4 (3)	10	24	26 (14)	50
	I	9	17 (12)	26	2	1 (1)	3	6	3 (2)	9	17	21 (15)	38
	% *B. microti-*infected	45	48.6 (57.1)	47.3	22.2	20 (33.3)	21.4	50	42.9 (40.0)	47.4	41.5	44.7 (51.7)	43.2
2014	NI	17	22 (0)	39	1	2 (0)	3	11	25 (3)	36	29	49 (3)	78
	I	16	14 (10)	30	0	0 (0)	0	14	7 (2)	21	30	21 (12)	51
	% *B. microti-*infected	48.5	38.9 (100)	43.5	0	0	0	56	21.9 (40.0)	36.8	50.9	30 (80)	39.5%
∑	NI	28	40 (9)	68	8	6 (2)	14	17	29 (6)	46	53	75 (17)	128
	I	25	31 (22)	56	2	1 (1)	3	20	10 (4)	30	47	42 (27)	89
	% *B. microti-*infected	47.2	43.7 (71)	45.2	20	14.3 (33.3)	17.7	54.1	25.6 (40.0)	39.5	47	35.9 (61.4)	41.0

*Abbreviations*: *NI* uninfected, *I* infected, in parentheses - no. of pregnant females

Among field voles, prevalence of *B. microti* was the lowest of all: 21.4% in 2013 and no *Babesia*-positive field voles were found among the three individuals trapped in 2014.

### Abundance of *B. microti* infection in the community of voles

Data on the abundance of *B. microti* infection by year of study, host species and sex is provided in Table 3. Abundance was calculated on the basis of microscopical observation of blood smears for 121 wild-caught *M. arvalis*, 76 *M. oeconomus* and 17 *M. agrestis*. The mean abundance of *B. microti* infection, calculated for the three vole species combined, was 15.33 ± 15.45 (19.99 ± 16.91 excluding *M. agrestis*) (Table 3).

**Table 3:** Abundance of *Babesia microti* (mean number of infected red blood cells (iRBC)/200 fields of vision ± standard error, SE) in wild-caught voles

	Year		
Species	2013	2014	Total
*M. arvalis*			
Males	58.38 ± 54.87	15.94 ± 48.85	37.16 ± 36.73
Females	10.32 ± 25.73	2.10 ± 30.68	6.21 ± 20.02
All	34.35 ± 30.30	9.02 ± 28.84	21.68 ± 20.92
*M. oeconomus*			
Males	32.32 ± 68.77	38.35 ± 48.12	35.93 ± 39.88
Females	0.00 ± 56.67	6.64 ± 35.63	3.32 ± 33.47
All	12.93 ± 43.74	22.49 ± 29.94	18.14 ± 25.73
*Microtus* spp^a^			
Males	47.95 ± 44.12	27.14 ± 35.26	36.60 ± 27.78
Females	5.16 ± 32.00	4.37 ± 24.17	4.76 ± 20.05
Overall mean	24.61 ± 26.59	15.75 ± 21.37	19.99 ± 16.91
*Microtus* spp.^b^			
Males	34.25 ± 34.16	23.27 ± 34.89	28.76 ± 24.41
Females	3.44 ± 29.30	3.74 ± 24.14	3.57 ± 19.58
Overall mean	16.920 ± 22.25	13.50 ± 21.22	15.33 ± 15.45

^a^Mean no. of iRBC/200 fields for combined *M. arvalis* and *M. oeconomus*

^b^Mean no. of iRBC/200 fields for three vole species including 17 individuals of *M. agrestis*

Mean abundance of *B. microti* was similar in *M. arvalis* and *M. oeconomus*, but no positive blood smears were identified among 17 *M. agrestis* (3 *Babesia*-positive samples by PCR only, Table 2) so the estimated mean abundance was close to zero for this host species. There were no significant differences in mean abundance of *B. microti* in the vole community between the years of study, host sexes and age classes (Table 3).

### Vertical transmission of *B. microti*

#### Prevalence of *B. microti* in females and dams

Altogether 117 female voles were trapped, among which 44 were pregnant thus providing 27 litters (embryos and pups) from *Babesia*-positive females and 17 litters from *Babesia*-negative mothers for analysis of vertical transmission (Fig. 1, Tables 2, 4 and 5). The overall prevalence of *B. microti* infection in the pregnant females was 61.4% (27/44). Highest prevalence was in pregnant female *M. arvalis* (71%, 22 litters from *Babesia*-positive females), 40% in pregnant female *M. oeconomus* (4 litters from *Babesia*-positive females) but only one female of 3 pregnant *M. agrestis* was found to be *Babesia-*positive (2013, 33.3%, 1 litter). There were significant differences in the prevalence of *B. microti* in pregnant females between host species and years of study (year × host species × *Babesia* infection: *χ* ^2^ = 12.10, *df* = 2, *P* = 0.002) (Table 2). All pregnant *M. arvalis* females trapped in 2014 were *Babesia*-positive (100%), in comparison with 57% *Babesia*-positive pregnant voles in 2013, but there were no differences in the prevalence of *B. microti* between years of study in pregnant female root voles (Table 2).

**Table 4:** Evidence for vertical transmission and genotype identity of *B. microti* in embryos isolated from female voles in 2013 and 2014

ID of pregnant female	Host species	No. of embryos in litter	No. of embryos infected with *B. microti* in the litter	% of infected embryos	*B. microti* genotype	
					In positive female	In embryos (no. of genotyped embryos)
2013/3	*M. arvalis*	7	7	100	IRU 1	IRU 1 (3)
2013/15	*M. arvalis*	4	4	100	IRU 1	IRU 2 (3)
2013/20	*M. arvalis*	5	5	100	IRU 1	IRU 2 (2)
2013/21	*M. arvalis*	6	6	100	IRU 1	IRU 2 (3)
2013/24	*M. agrestis*	7	0	0	IRU 1	nd
2013/37	*M. arvalis*	5	5	100	IRU 1	IRU 2 (2)
2013/41	*M. arvalis*	6	6	100	nd	IRU 2 (3)
2013/47	*M. arvalis*	2	2	100	IRU 1	IRU 1 (1)
2013/52	*M. arvalis*	6	6	100	IRU 2	IRU 1 (1) and IRU 2 (2)
2013/53	*M. arvalis*	6	6	100	nd	IRU 2 (1)
2013/63	*M. oeconomus*	5	5	100	IRU 2	IRU 2 (1)
2013/72	*M. arvalis*	6	6	100	IRU 1	IRU 1 (2)
2014/44	*M. oeconomus*	6	0	0	IRU 1	nd
2014/155	*M. arvalis*	4	3	75	nd	IRU 2 (3)
Total		75	61(81.3%)	Litters positive 12/14 (85.7%)	9 × IRU 1; 2 × IRU 2	7 × IRU 1; 20 × IRU 2

*Abbreviation*: *nd* not done

**Table 5:** Evidence for vertical transmission and genotypes of *B. microti* in pups delivered by female voles captured in 2014

ID of pregnant female	Host species	No. of pups in a litter	No. of embryos infected with *B. microti* in the litter	% of infected pups	*B. microti* genotype	
					In positive dam	No. of pups (no. of genotyped pups)
2014/25	*M. arvalis*	6	5	83	IRU 1	IRU 1(1) and IRU 2 (1)
2014/34	*M. arvalis*	5	4	80	IRU 2	IRU 1 (2)
2014/59	*M. arvalis*	5	5	100	nd	IRU 1 (1) and IRU 2 (1)
2014/65	*M. arvalis*	6	6	100	IRU 1	IRU 1 (1) and IRU 2 (1)
2014/77	*M. oeconomus*	6	5	83	IRU 2	IRU 2 (2)
2014/107	*M. arvalis*	6^a^	0	0	IRU 1	nd
2014/112	*M. arvalis*	5	5	100	nd	IRU 2 (1)
2014/126	*M. arvalis*	7	4	57	nd	IRU 2 (1)
2014/130	*M. arvalis*	4	3	75	nd	IRU 2 (3)
2014/131	*M. arvalis*	6	3	50	nd	IRU 2 (2)
In total		56	40 (71.4%)	Litters positive 9/10 (90%)	3 × IRU 1; 2 × IRU 2	5 × IRU 1; 12 × IRU 2

*Abbreviation*: *nd* not done

^a^Pups died after birth

Of the 44 pregnant females, 11 were kept in captivity until pup delivery, and these provided 10 litters from *Babesia*-positive females (host species and litter size provided in Table 5) and 1 litter (6 pups) from a *Babesia*-negative *M. oeconomus* female (Fig. 1). Reliable analysis of the prevalence of infections in embryos was possible for 113 embryos from another 20 litters [14 litters from *Babesia*-positive females (Fig. 1, Table 4) and 6 litters from *Babesia*-negative females]. These embryos were of an appropriate size to enable autopsy and isolation of organs (heart with lungs, for all samples). In the remaining 13 cases of pregnancy (3 *Babesia*-positive females and 10 *Babesia*-negative females), pregnancies were at an early stage and no reliable isolation of embryos’ organs could be carried out.

#### Detection of B. microti in pregnant females and embryos (2013 and 2014)

Prevalence of *B. microti* infection as determined by PCR and nested PCR among the 113 embryos of the 20 terminally euthanized females was 70% (14 litters and 75 embryos from *Babesia*-positive females and 6 litters and 38 embryos from *Babesia*-negative females). Among *Babesia*-positive pregnant females, 11 were *M. arvalis*, two *M. oeconomus* and one *M. agrestis* (Fig. 1, Table 4). *Babesia*-positive tissues (heart and lungs) in embryos were found in 85.7% (12/14) of these litters. No *B. microti* DNA was detected in 38 embryos of the 6 *Babesia*-negative females (2 *M. arvalis*, 3 *M. oeconomus*, 1 *M. agrestis*), in comparison to 61 positive of 75 embryos recovered from 14 *Babesia*-positive females (81.3%) (Fisher’s exact test, *P* < 0.0001). In addition to the *Babesia* positive heart and lung samples, the DNA of *B. microti* was detected also in liver tissues in 4 out of 5 tested embryos (*M. arvalis*).

Among the three host species, no *B. microti* DNA was detected in 7 embryos from 1 litter of one *Babesia*-positive *M. agrestis* female, in comparison to 56/57 positive embryos from 11 *M. arvalis* females (vertical transmission confirmed in all litters with overall 98% embryos positive for *B. microti*, and between 75–100% success of transmission per litter; Table 4). Among two *Babesia*-positive *M. oeconomus* females, *B. microti* DNA was detected in all 5 embryos from one litter and in none of 6 embryos of a second litter, giving in total 50% success of vertical transmission for litters and 46% of *Babesia-*positive embryos from infected females (Table 4). In summary, *B. microti* DNA was detected in 0, 50 and 100% of the litters of *Babesia*-positive *M. agrestis*, *M. oeconomus* and *M. arvalis* females, respectively. Among these litters, 0, 46 and 98% of embryos were infected with *B. microti* for *Babesia*-positive *M. agrestis*, *M. oeconomus* and *M. arvalis* females, respectively, and these differences in the success of vertical transmission of *B. microti* were statistically significant (host species × *Babesia* presence/absence in embryos: *χ* ^2^ = 51.28, *df* = 2, *P* < 0.0001).

#### Detection of *B. microti* in dams and pups maintained in vector-free conditions (2014)

In the second year of the study, 11 pregnant females (9 *M. arvalis* and 2 *M. oeconomus*), deprived of all ectoparasites, were kept in our animal house until they had given birth and weaned their pups (*n* = 62). *Babesia microti* DNA was detected in all *M. arvalis* dams and in one of two *M. oeconomus* dams (Table 5). No *B. microti* DNA was detected in 6 pups delivered by a *Babesia*-negative *M. oeconomus* dam, in comparison to 40/56 (71.4%) positive pups delivered by 10 *Babesia*-positive dams. In one litter from a *Babesia*-positive *M. oeconomus* dam, 5 of 6 pups were positive (83%), in comparison to 70% (35/50) positive pups from 9 *Babesia*-positive *M. arvalis* dams (Table 5) (difference not significant, NS). Among 9 litters from *M. arvalis* dams, *Babesia*-positive pups were found in 8 litters (8/9 litters i.e. 89% of success in litters) and among positive litters, the percentage of *Babesia*-positive pups varied in the range 50–100% (Table 5).

The percentage of *Babesia*-positive pups in a litter was negatively correlated with litter size (*r*_*S*_ = -0.661, *P* = 0.052) (Table 5). There was no significant difference between male and female pups born from infected dams: 86.2% of males and 71.4% of females were infected with *B. microti*.

When we analyzed data on embryos and pups together, the significant factors influencing *Babesia* infection in offspring were: host species (host species × *Babesia* presence/absence in embryos/pups: *χ* ^2^ = 46.43, *df* = 2, *P* < 0.0001) with the highest success of vertical transmission in *M. arvalis* as described above; infection in the mother (*χ* ^2^ = 84.30, *df* = 1, *P* < 0.0001) with no infections in the offspring of *Babesia*-negative females and a high rate of congenital infections in offspring of *Babesia*-positive females (Tables 4 and 5); and year of study (year × *Babesia* presence/absence in embryos/pups: *χ* ^2^ = 29.99, *df* = 1, *P* < 0.0001). Interestingly, a higher percentage of *Babesia*-positive offspring was obtained in the first year of the study when we focused on embryos, in comparison to 2014, when the focus was on pups (Tables 4 and 5).

#### Influence of congenitally acquired *B. microti* infection on litter size, body mass and survival of pups

Two litters (6 pups of *M. arvalis* and 6 pups of *M. oeconomus*) died 1–2 days after birth. All these pups were *Babesia*-negative by PCR, although one litter was delivered by a *Babesia*-positive dam (*M. arvalis*, ID 2014/107; Table 5). The other litter was delivered by the only one *Babesia*-negative *M. oeconomus* dam. All the other pups delivered by 9 *Babesia*-positive dams (40 *Babesia*-positive and 10 *Babesia*-negative pups; Table 5) survived until the end of the experiment. Thus the mortality of pups was 0% among *Babesia*-positive and 54.5% (12/22) among *Babesia*-negative pups and this difference was significant (alive/dead pup × *Babesia* presence/absence: *χ* ^2^ = 30.61, *df* = 1, *P* < 0.0001).

The mean litter size for all 11 dams was 5.85 ± 0.43 and was similar among *M. arvalis* and *M. oeconomus* dams (5.56 ± 0.29 and 6.0 ± 0.62; NS). The effect of *Babesia* infection in the dam on the litter size could not be reliably analyzed as there was only one litter from a *Babesia*-negative dam (with 6 pups) and the mean litter size for *Babesia*-positive dams was 5.78 ± 0.47 (NS).

The mean body mass of *M. oeconomus* pups at age of 3 weeks was significantly higher than for *M. arvalis* pups: 17.86 ± 1.09 g and 15.13 ± 0.46 g, respectively (main effect of host species on body mass of pups: *F*_(1,49)_ = 4.78; *P* = 0.03). Male pups of *M. arvalis* were slightly heavier (15.80 ± 0.66 g) than females (14.45 ± 0.64 g), but for *M. oeconomus* pups the mean weight of pups was closer: 17.75 ± 1.71 g for males and 17.92 ± 1.40 g for females (NS). The mean weight was almost identical for *Babesia*-positive pups and *Babesia*-negative pups (16.59 ± 0.59 g and 15.91 ± 0.97 g) (NS).

The abundance of *B. microti* was calculated on the basis of microscopical observation of blood smears of 44 *M. arvalis* and 6 of *M. oeconomus* pups*.* The mean abundance of *B. microti* in blood smears collected from offspring of infected dams was 0.54 ± 0.11, but this was twice as high in *M. oeconomus* compared with *M. arvalis* pups (0.75 ± 0.21 and 0.32 ± 0.74, respectively; *F*_(1,49)_ = 3.78, *P* = 0.06).

#### Genotyping of *B. microti* isolates from wild-caught voles and congenitally acquired infections

Altogether 97 (73 *M. arvalis*, 22 *M. oeconomus* and 2 *M. agrestis*) *Babesia* sequences were obtained. Among these, 53 were derived from naturally infected voles, including pregnant females and dams (32 *M. arvalis*, 19 *M. oeconomus* and 2 *M. agrestis*) and 44 were obtained from embryos or pups.

Alignment of the sequences revealed that two main *B. microti* genotypes were found in wild-caught voles, pregnant females and embryos, dams and their pups: one genotype was most similar (98–100% of similarity) to *B. microti* IRU1 isolate (KC470048), closely related to the pathogenic Jena strain (EF413181) isolated from human blood [31] and the second genotype was most similar (98–100%) to the *B. microti* IRU2 isolate (KC470049), closely related to the non-pathogenic Munich strain (AY789075), first isolated from the house mouse *Mus musculus* by Tsuji and Ishihara (2001, published on GenBank only). Lower similarity for several sequences was the result of some non-specific background amplification of DNA. Both IRU1 and IRU2 genotypes of *B. microti* have been detected previously in *I. ricinus* ticks in our earlier studies in the same region of Poland [10].

The IRU1 (Jena-like) *B. microti* genotype was dominant among wild-caught voles (49/53; 92%), pregnant females (81.8%) and dams (60%) and altogether was identified in 62.9% (61/97) of the sequenced isolates. The IRU2 (Munich-like) genotype was dominant among positive embryos (74.1%) and pups (70.6%) and altogether was identified in 37.1% (36/97) of the *Babesia* isolates (Tables 4 and 5).

Among the *B. microti* sequences obtained from wild-caught voles, 32 were from *M. arvalis*, 19 from *M. oeconomus* and 2 from *M. agrestis*. The *B. microti* IRU1 genotype (Jena-like) was identified in 94% of sequences derived from *M. arvalis* (10 from males and 20 from females), in 89% of sequences derived from *M. oeconomus* (13 from males, 4 from females) and in both sequences from *M. agrestis* (1 from a male and 1 from a female vole). The IRU2 genotype of *B. microti* (Munich-like) was identified in 4 isolates from females (2 *M. arvalis* and 2 *M. oeconomus*).

The final step of our study on vertical transmission was to determine the *B. microti* genotype infecting females/dams and their embryos/pups. We were able to sequence eleven PCR products from pregnant females (Table 4: 8 from *M. arvalis*, 2 from *M. oeconomus* and 1 from *M. agrestis*) and selected 27 embryos recovered from these females. In 3 cases the genotypes of *B. microti* identified in the female and her offspring were identical (IRU1 genotype) and in one case either the IRU1 or IRU2 genotypes were found in isolates from offspring. In 4 cases the *B. microti* genotype identified in the female was different from the genotype identified in the embryos (Table 4: the *B. microti* IRU1 genotype in females but IRU2 in embryos). Thus, the dominant *B. microti* genotype identified in pregnant females was IRU1 (9/11; 81.8%). In embryos, the IRU2 genotype was identified more often (20/27; 74.1%). Interestingly, for two females infected with the *B. microti* IRU1 (Jena-like) genotype strain (1 *M. oeconomus* and 1 *M. agrestis*) no evidence of vertical transmission in embryos was found (all embryos were *Babesia*-negative).

In 2014 we were able to sequence PCR products from 5 dams (4 *M. arvalis* and 1 *M. oeconomus*) and from selected pups of 9 dams (Table 5). In one case the genotype of *B. microti* identified in the dam and her two pups was identical (IRU2 genotype) and in 2 cases either the IRU1 or IRU2 genotypes were found in isolates from pups. In one case the *B. microti* genotype identified in the dam was different from the genotype identified in the pups (Table 5: *B. microti* IRU2 genotype in dam but the IRU1 genotype in two pups). In four other litters, where the *B. microti* genotype in the dams could not be determined, the IRU2 genotype was identified in pups, and in one litter again both *B. microti* genotypes were found (Table 5). Thus, the dominant *B. microti* genotype identified in dams was IRU1 (3/5, 60%) whereas among pups, the IRU2 genotype was more common (12/17; 70.6%).

## Discussion

In this study we reported a high prevalence of *B. microti* in a *Microtus* spp. community in Poland and provided evidence in support of the idea that, in two main host species, *M. arvalis* and *M. oeconomus*, high prevalence can be partially maintained by a high rate of vertical transmission from naturally infected female voles to their offspring. We also reported a complex circulation of two main rodent *B. microti* genotypes, the zoonotic Jena-like (IRU1) and the enzoonotic Munich-like (IRU2) genotypes, in the community of three *Microtus* species.

Although the present study focused primarily on the occurrence of vertical transmission of *B. microti* in the three vole species, it also provided novel data to complement our interest in the long-term dynamics of *B. microti* at our study sites in the Mazury Lake District. The first study on *B. microti* in voles was carried out in 1997–2000 [1] and focused on *M. arvalis*; then in 2004–2006 the second study incorporated *M. arvalis* and *M. oeconomus* populations [2, 34] and finally, in the present paper we report on *B. microti* prevalence in the community of three vole species. Overall prevalence of *B. microti* in this period of 17 years was lowest in the first 4 years (9%; [1]) and was similar in two latter surveys (32–35% [2] versus 41% in the present report). However, the markedly lower prevalence of *B. microti* in *M. arvalis* in the first study is probably attributable mostly to a different sampling strategy and detection techniques - the study was spread over three seasons of the year (spring, summer and autumn) and based solely on microscopical observation of blood smears, which is a far less sensitive method for the detection of chronic infections of *B. microti* in comparison to molecular techniques, as demonstrated in the experimental study by Welc-Faleciak et al. [35]. Building on this first study, where *B. microti* infections in voles were apparently seasonal, with maximum prevalence in summer, the two latter studies were carried out only in summer months (August and early September) and employed molecular techniques (PCRs) for detection of the parasite, thus providing more comparable data over the period of ten years from 2004 to 2014. Prevalence of *B. microti* was highest in *M. arvalis* in this period (35–45%) and only slightly lower in *M. oeconomus* (32–40%). A similar pattern was observed in abundance of *B. microti*. Interestingly, both parameters were lowest in the third species, *M. agrestis*, which was sampled and studied only in 2013–2014. This species is rarely reported from our study sites in the Mazury Lake District [36] and as our data show, this is a species of much lower significance as a reservoir host of *B. microti*. Interestingly, over the long-term, there were a few years when *B. microti* prevalence in *M. arvalis* was extremely high, as for example exceeding 20–50% across three seasons in 1998 or 70% in the summer months of 2005 [1, 34]. Our finding that *M. arvalis* and *M. oeconomus* are the principal reservoir hosts of *B. microti* is supported by other studies from north-eastern Poland and from other regions of central Europe [37–42]. In Poland, prevalence has been reported in the range of 9–72% for *M. arvalis* and 8–50% for *M. oeconomus* [4, 37, 38] and within the range of 0.6–14% in other countries [39–42]. Surveys in the UK, where *M. agrestis* is the only species of the genus *Microtus* and is reported as the main host of *B. microti,* have reported high prevalence values within the range of 22–30%, higher than in our study sites [43–46]. High prevalence of *B. microti* in *M. agrestis* has been reported also in Southern Poland (50% in Katowice; [37]), Germany (38%; [47]), Austria (31%; [48]) and Russia (52%, [14]). The overall prevalence in the community of voles in the current study was similar to prevalence in Omsk region, Russia (31.6%, [14]).

We found intriguing the generally low infestation of *I. ricinus* ticks, hosts of *B. microti*, on *Microtus* spp. and the high prevalence of the parasite in voles, in contrast to the high infestation of *I. ricinus* ticks on woodland rodents and the generally low prevalence of *B. microti* in the latter hosts. Therefore we tested the hypothesis that high prevalence of *B. microti* in *Microtus* hosts maybe achieved by vector independent vertical transmission of parasites between females and their offspring. Quite clearly our observations, whether based on pregnant females-embryos or dams-pups, support our hypothesis, both revealing a high rate of vertical transmission in *M. arvalis* and *M. oeconomus*. Altogether 81% of embryos from *Babesia*-positive females and 71% of pups from *Babesia*-positive dams were *Babesia*-positive. This rate of *Babesia*-positive offspring derived from *Babesia*-positive female voles may be compared with an overall prevalence of *B. microti* in juvenile voles of 19% (in juveniles of all species combined; 25% of juveniles of *M. arvalis*). However, to enable a more meaningful comparison, estimation of *Babesia*-positive offspring should include also *Babesia*-negative offspring of *Babesia*-negative females. Combing these data, we obtain a value of 58% for the prevalence of *Babesia* in the offspring in the F1 generation (2013 and 2014), which is higher than the prevalence observed in wild-caught juvenile voles. This difference may be explained by two mechanisms - the progressive loss of congenitally acquired infection with age (which explains also the difference between the percentages of *Babesia*-positive embryos and pups) and/or faster loss of infected offspring under natural conditions, i.e. by predation. To support the latter hypothesis (on the negative impact of congenitally acquired *Babesia* infection), we compared selected parameters between *Babesia*-positive and *Babesia*-negative litters (litter size) and pups (i.e. survival rate, mean body weight). However, no evidence was found to support this hypothesis, as mean litter size and body weight were almost identical in both groups. In fact, in contrast to our expectations, the survival rate over three weeks after birth was lower among *Babesia*-negative pups. These findings support the ‘balancing strategy hypothesis’ [49]. The balancing strategy hypothesis proposes that long-term co-evolution of parasite-host interactions results in a ‘balanced’ system, with a low negative impact of parasites on the host population, low pathogenicity and mortality enabling simultaneous propagation of both parasite and host without epidemic periods that are characteristic in many viruses and bacteria systems which follow an ‘opposing strategy’. The very low parasitaemia found in the pups with congenitally acquired *B. microti* infection (1–5 iRBC/200 fields of vision) in comparison to wild-caught voles (mean19.99 ± 16.91 iRBC/200 fields) supports this hypothesis, together with the known long-term survival of *B. microti* infection in rodent hosts under natural and experimental conditions [15, 35]. Thus *Babesia* may be considered to be a master of a balancing strategy, together with *Plasmodium falciparum*, given as the example by Wenk & Renz [49].

The occurrence of *Babesia*-positive litters and *Babesia*-positive offspring was higher in *M. arvalis* than in *M. oeconomus*, reflecting a slightly but permanently higher prevalence of *B. microti* in common voles throughout the 17-year-long period of field studies in the Mazury Lake District [1, 2, 50]. Interestingly, we observed lower success of vertical transmission (% of *Babesia*-positive) in larger litters of pups in comparison to smaller, and this may represent a ‘dilution effect’, described for some parasite species in high-density populations of their hosts [51].

The final steps to complete the study on vertical transmission were to identify the genotypes of *B. microti* in the community of voles, in pairs of females and their offspring, and to determine the prevalence of zoonotic to non-zoonotic strains in both mothers and their offspring. In the event, a complex picture emerged, involving two common strains of *B. microti.* Interestingly, both *B. microti* strains, the zoonotic Jena-like (IRU1) and non-pathogenic Munich-like (IRU2) genotypes were found in both wild-caught voles and the captive-maintained female-offspring group. The Jena-like strain was dominant among wild-caught voles, including pregnant females and dams. These results correspond to our earlier results during the period 2004–2006 [32]. In 2004, all the *B. microti* isolates that were sequenced were identified as the Munich-like strain and this strain was involved in an ‘outbreak’ of *B. microti* infection in *M. arvalis*, with prevalence exceeding 70% [32]. In 2005 again this strain was dominant, with a low percentage of isolates identified as the Jena-like strain, but in 2006 the pattern was reversed with all genotyped isolates identified as the Jena-like strain. Also in Russia, the enzootic *B. microti* strain (Munich) and the zoonotic strain (US/Gray/Jena strain) have been fund to be sympatric in different study sites, but with dominance by one or the other strain depending on the location [14, 52, 53]. The dominant strain in the region of Omsk was *B. microti* Munich with a dominance of 93% [14], in contrast to the 92% for *B. microti* IRU1/Jena-like stain in our study. However, in the present study the picture appears to be more complex because of the occurrence of different strains in the female-offspring combinations and also the detection of both strains in a single litter. Our results show that mixed infections of both genotypes can occur in adult voles, with the Jena-like (IRU1) genotype more ‘detectable’ or dominant in adult voles and the Munich-like (IRU2) genotype more detectable or dominant in offspring. One possibility is that the Munich-like strain has a better capacity for vertical transmission and propagation in younger voles, while the Jena-like strain may be better at maintaining a chronic infection in voles but has a lower success in vertical transmission (Tables 4 and 5). These proposed contrasting biological traits of the *B. microti* IRU1 and IRU2 strains may be responsible for the dynamic pattern in the proportion of each strain observed in our study sites throughout the years over which we have monitored the host populations. However, the possibility that the two strains differ in pathogenicity requires further study, especially regarding the zoonotic significance of the Jena-like strain.

## Conclusions

A high rate of vertical transmission of two main genotypes of *B. microti* has been confirmed in two species of naturally infected voles, *M. arvalis* and *M. oeconomus*, resulting in an overall high prevalence of infection in a community of *Microtus* spp. voles from nort-eastern Poland.
